# Systemic ALK-positive anaplastic large cell lymphoma involving implant site: a fortuitous association

**DOI:** 10.4322/acr.2021.296

**Published:** 2021-08-20

**Authors:** Mayur Parkhi, Charanpreet Singh, Rajender Kumar, Pankaj Malhotra, Amanjit Bal

**Affiliations:** 1 Post Graduate Institute of Medical Education and Research, Department of Histopathology, Chandigarh, India; 2 Post Graduate Institute of Medical Education and Research, Department of Clinical hematology, Chandigarh, India; 3 Post Graduate Institute of Medical Education and Research, Department of Nuclear Medicine, Chandigarh, India

**Keywords:** Femoral Fractures, Prostheses and Implants, Lymphoma, Large-Cell, Anaplastic, Anaplastic Lymphoma Kinase

## Abstract

Anaplastic lymphoma kinase (ALK) positive, anaplastic large cell lymphoma involving the non-mammary implant is an extremely rare presentation. Irrespective of the type or site, the implant-associated primary ALCL is morphologically and immunophenotypically similar to ALK-negative ALCLs. Herein, we present the case of a 42-year-old male who developed a lytic lesion after an implant for a right femur fracture. The lytic lesion biopsy revealed anaplastic large cell lymphoma with ALK protein expression. Imaging findings showed the widespread dissemination of disease all over the body, entrapping the implant too. ALCL involving the bone implant is a very unusual and rare presentation that needs to be documented.

## INTRODUCTION

Anaplastic large cell lymphoma (ALCL) accounts for 5% of all non-Hodgkin’s lymphomas^1^. Stein^2^ was the first to describe this entity based on the proliferation of large pleomorphic cells that expressed CD30. Although systemic ALCL frequently involves the bone marrow, the primary or secondary involvement of bone is infrequent and rare appearance. In the literature, there are few registered case reports and small case series of ALK-positive ALCL showing bone involvement.^3^^,^ 4 On the contrary, ALCL involving the bone implant is very unusual, and uncommon. Breast implant-associated anaplastic large cell lymphoma (BIA-ALCL), first reported by Keech and Creech^5^, was enrolled as a provisional entity in the 2017 edition of the WHO classification of hematopoietic and lymphoid neoplasms.^6^ Moreover, three case reports of non-mammary implant-associated anaplastic large cell lymphoma (NMIA-ALCL) have been documented in the last decade.^7^^-^ 9 Akin to BIA-ALCL, the non-mammary implant-associated ALCL (NMIA-ALCL) showed morphologic and immunophenotypic features of ALK-negative anaplastic large cell lymphoma. We discuss the case of a 42-year-old male with a history of a road traffic accident and fracture of the right femur, which was followed by open reduction and internal fixation (ORIF). Almost two decades later, the patient developed an ALK-positive anaplastic large cell lymphoma involving the implant site along with systemic involvement. The index case of systemic ALCL, showing involvement of metal rod implant, is an exceedingly rare chance and thus, warrants documentation in the literature.

## CASE REPORT

**Figure 1:**
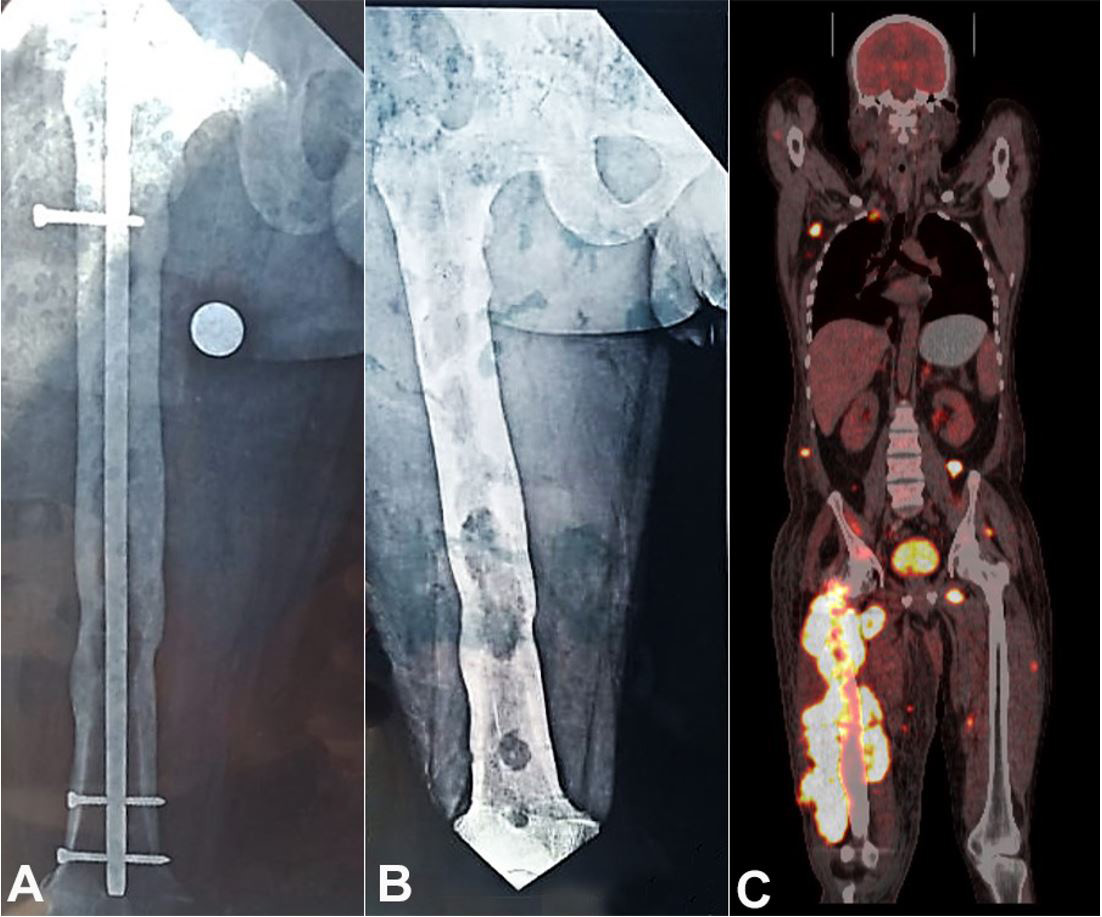
A and B – Antero-posterior and lateral plain radiographic view of the right lower extremity showing a femur with and without metallic rod and fixation screws. The ill-defined, expansile, intramedullary lytic lesions are seen involving metaphyseal and diaphyseal regions. C – Coronal fused PET/CT showing increased FDG uptake as the large skeletal lesion involving right femur and few enlarged lymph nodes along with subcutaneous and intramuscular deposits.

A 42-year-old man presented with the complaint of painful swelling in the right lower limb for 5 months. Also, he noted a serous discharge from the scar of the previous surgery, over the last 5 months. His medical history included an internal fixation with a metal rod implant for femoral fracture treatment (Figure 1A). Roughly two months after the initial symptoms, the implant was removed (Figure 1B). The computed tomography (CT) detected a huge right proximal thigh lesion as well as multiple soft tissue lesions. These soft tissue lesions included the subcutaneous tissue of the face and abdominal wall, left perinephric and posterior pararenal space, left gluteal region, right second rib, and the right iliac bone. The Magnetic resonance imaging (MRI) scan of the right thigh showed an ill-defined, lobulated, expansile, intramedullary lytic lesion, which involved the metaphyseal and diaphyseal region of the femur. It also showed thickening and multi-focal breach of the cortex with associated extraosseous soft tissue component involving the skeletal muscle tissue. The positron emission tomography (PET) showed increased FDG avid uptake in the right thigh, nodal and extranodal tissue, intramuscular and subcutaneous region, renal, bowel, skeletal, omental, and peritoneal region, indicating the possibility of a disseminated malignancy (Figure 1C).

A biopsy of the femoral lytic region was performed, and the patient underwent a prophylactic internal fixation of the right femur with nails.

**Figure 2:**
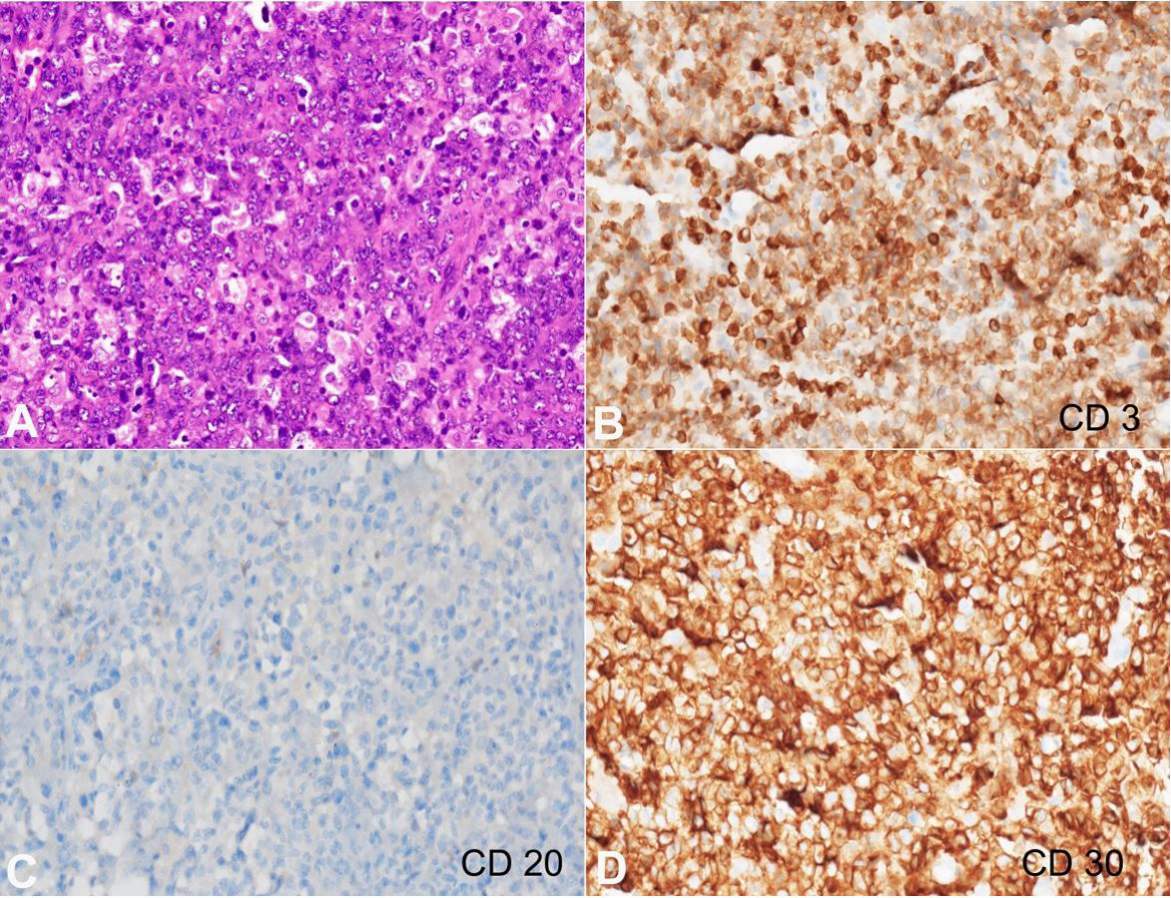
Photomicrographs of the biopsy. A – Large, atypical cells seen in solid sheets showing nuclear indentation, vesicular chromatin, prominent nucleoli and moderate basophilic to eosinophilic cytoplasm (H&E; x200); B – CD3 immunostain showing membranous positivity of variable intensity (x200); C – CD20 immunostain is negative (x200), D - CD30 displays strong membranous and golgi complex immunoreactivity (x200).

**Figure 3:**
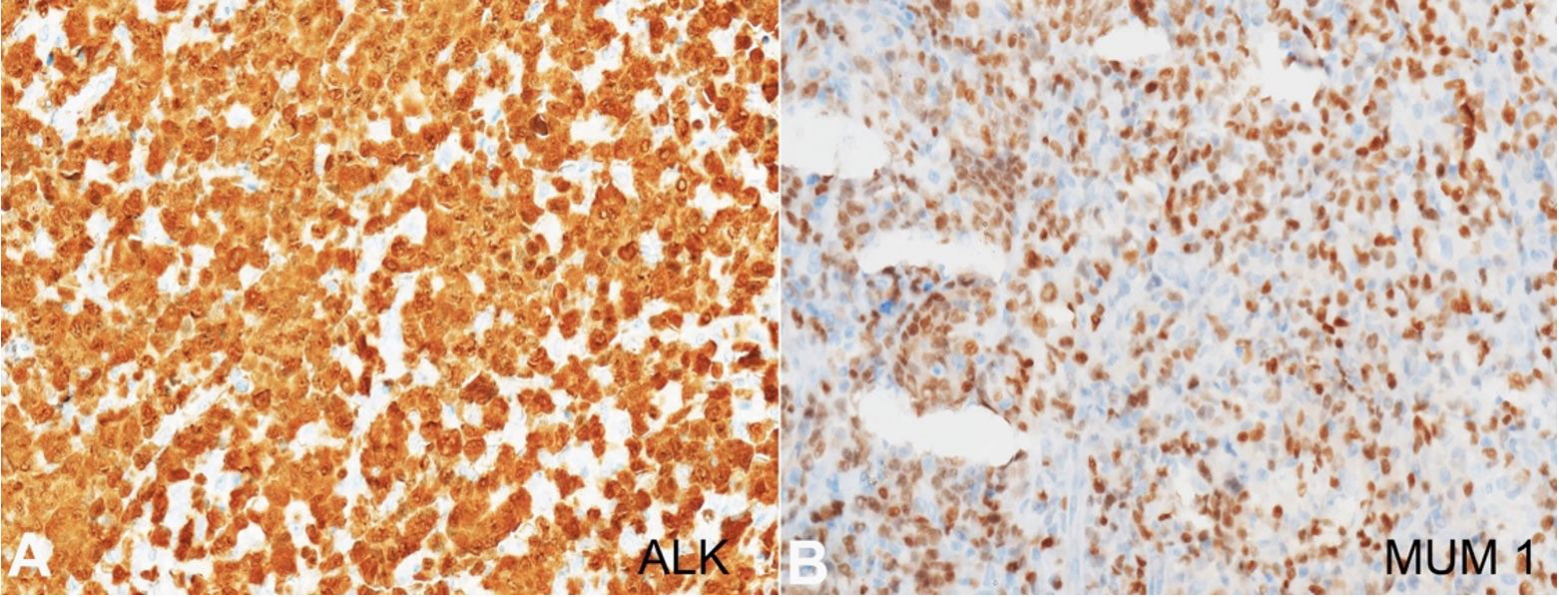
Photomicrographs of the biopsy. A – ALK immunostain showing diffuse positivity in both nuclear and cytoplasm (x200); B – MUM1 showing nuclear positivity (x200).

The biopsy’s histology revealed large, atypical cells in solid sheets with a round to oval irregular nuclei and nuclear indentations (Figure 2A). The chromatin was coarse to vesicular with prominent nucleoli and moderate basophilic to eosinophilic cytoplasm. Amidst these large cells, the characteristic hallmark cells with kidney-shaped nuclei were also present. Mitotic activity was brisk. These neoplastic cells were invading and replacing the surrounding bone trabeculae, indicated by dead bony fragments, collection of giant osteoclast cells, and reactive bone formation. Besides, the skeletal muscle and fibro-adipose tissue involvement hinted at the extraosseous presence. On immunohistochemistry (Figure 2B-D and 3A, B), the Leukocyte common antigen (LCA) showed membranous positivity of variable intensity, and the CD20 immunostain was negative. CD3 showed weak membranous positivity, and CD30 showed diffuse, strong, and uniform immunoreactivity in the large cells in the membrane and Golgi zone. ALK immunostain revealed diffuse nuclear as well as cytoplasmic positivity. MUM1 depicted strong, diffuse, nuclear immunostaining. CD2, CD4, CD5, CD7, CD8, CD138, c-MYC, P63 and pan-cytokeratin were negative. In-situ hybridization for Epstein Barr virus was negative. On histopathology, a diagnosis of anaplastic large cell lymphoma, ALK-positive, was entertained. Taken together with the clinical presentation, imaging details and histopathological findings, this case seems more to be systemic ALCL with widespread dissemination with secondary involvement of the metal rod implant.

The patient received one cycle of chemotherapy in the form of CHOEP (Cyclophosphamide, Doxorubicin, Vincristine, Etoposide, and Prednisone) regime following the diagnosis. Unfortunately, he died as a result of therapy-related complications.

## DISCUSSION

Anaplastic large cell lymphoma (ALCL) with primary or secondary involvement of bone is a rare presentation. The index case showed ALCL at the implanted prostheses along with widespread dissemination involving the soft tissue, nodes, and multiple bones. The sequence of appearance was not clear, and thus, the primary source as nodal or extranodal in this case was difficult to determine. However, the probability of systemic ALCL was considered based on clinical, radiological, and histopathological correlation. The reason behind documenting this case was secondary involvement of the implant in the setting of systemic ALCL, which is a very rare and interesting finding; hardly mentioned in the literature.

Among the extranodal non-Hodgkin lymphomas (NHL) in association with metallic prostheses or osteosynthetic materials, large B-cell lymphomas comprise the majority, while the T-cell lymphoma (i.e., anaplastic large cell lymphoma) constitutes a handful of cases.^7^^-^ 11 Curiously, our patient had undergone a metal rod implant for femur fracture, two decades prior. Similar to the breast implant-associated ALCL (BIA-ALCL), non-mammary implant associated anaplastic large cell lymphoma have morphological and immunophenotypic features indistinguishable from ALK-negative ALCL.^3^^-^ 5 ALK-positive ALCL, has not been reported in association with orthopedic prostheses or with breast implants to the best of our knowledge. The positive staining of ALK in the nucleus and cytoplasm corroborates with the presence of classic t(2;5)(p23;q35) translocation of ALCL.^9^ Comparable ALK staining pattern was evident in our case and was positive diffusely in the lymphoma cells.

The ALK-positive ALCL carries a better prognosis in comparison to the ALK-negative counterpart. However, the concurrent MYC rearrangement or histological variant (small cell or lymphohistiocytic) of ALK-positive ALCL are more prone for the aggressive course rather than favorable outcome.^6^ Besides, the work-up for the rearrangements (TP63 and DUSP22) were not required in the present case, which are exclusively seen in ALK-negative ALCLs. BIA-ALCL despite being ALK negative are known to have an excellent prognosis, as most of them are restricted to seroma cavity, and present as stage I disease. Very infrequently, BIA-ALCLs are associated with disseminated disease.^6^ Similarly, non-mammary implant-associated ALCL reported to date were localized to the implant site, lacked systemic spread, and showed excellent prognosis.^7^^-^ 9 In contrast, the index case demonstrated widespread dissemination of disease with involvement of implant site, which could be a chance association, difficult to explain. The uncertain hypothesis considered for the pathogenesis of implant-associated ALCL includes chronic inflammation (implant acting as antigenic stimulus) or implant surface (textured are more prone than smooth surface) that contributes for lymphomagenesis, cannot be employed here in the index case.

The majority of ALK-positive ALCL patients are expected to be cured with frontline chemotherapy, whereas the relapsed/refractory ones can be treated using therapeutic options such as crizotinib, anti-CD30 (brentuximab vedotin) or allogeneic bone marrow transplantation.^12^ The index patient received one cycle of chemotherapy (CHOEP regime: cyclophosphamide, doxorubicin, etoposide, vincristine, and prednisone), but died, after one cycle of CHOEP due to therapy-related complications. The majority of the patients diagnosed with BIA-ALCL have excellent outcomes after complete excision of the implant alone. A similar prognosis was evident among reported cases of non-mammary implant-associated ALCL, although chemotherapy option was considered in one of them.^7^^-^ 9

In conclusion, we have come across ALCL involving the non-mammary implant site associated with orthopedic prostheses. However, the positive expression of ALK protein and aggressive clinical course with a widespread disease in the present case made us re-evaluate it with a better understanding of clinico-radio-pathological correlation. This is an exceedingly rare and thought-provoking finding of the fortuitous involvement of the prostheses (i.e., metal rod implant) in the setting of systemic ALK-positive ALCL.
